# Polysaccharides from *Basella alba* Protect Post-Mitotic Neurons against Cell Cycle Re-Entry and Apoptosis Induced by the Amyloid-Beta Peptide by Blocking Sonic Hedgehog Expression

**DOI:** 10.3390/ijms25137316

**Published:** 2024-07-03

**Authors:** Bo-Yu Hou, Ming-Hsuan Wu, Hui-Yu Hsu, Yi-Chun Lin, Ding-I Yang

**Affiliations:** 1 Institute of Brain Science, National Yang Ming Chiao Tung University, Taipei 112304, Taiwan; tkoleo84119@gmail.com (B.-Y.H.); mission54321zack@gmail.com (M.-H.W.); 2 Mynature Biotech Inc., Yilan 260021, Taiwan; michellehsu1118@gmail.com; 3 Department of Neurology, Taipei City Hospital Renai Branch, Taipei 106243, Taiwan; 4 Brain Research Center, National Yang Ming Chiao Tung University, Taipei 112304, Taiwan

**Keywords:** Alzheimer’s disease, 5-bromo-2′-deoxyuridine (BrdU), caspase-3, cyclin D1, histone H3, mitochondria, proliferating cell nuclear antigen (PCNA)

## Abstract

The amyloid-beta peptide (Aβ) is the neurotoxic component in senile plaques of Alzheimer’s disease (AD) brains. Previously we have reported that Aβ toxicity is mediated by the induction of sonic hedgehog (SHH) to trigger cell cycle re-entry (CCR) and apoptosis in post-mitotic neurons. *Basella alba* is a vegetable whose polysaccharides carry immunomodulatory and anti-cancer actions, but their protective effects against neurodegeneration have never been reported. Herein, we tested whether polysaccharides derived from *Basella alba* (PPV-6) may inhibit Aβ toxicity and explored its underlying mechanisms. In differentiated rat cortical neurons, Aβ25-35 reduced cell viability, damaged neuronal structure, and compromised mitochondrial bioenergetic functions, all of which were recovered by PPV-6. Immunocytochemistry and western blotting revealed that Aβ25-35-mediated induction of cell cycle markers including cyclin D1, proliferating cell nuclear antigen (PCNA), and histone H3 phosphorylated at Ser-10 (p-Histone H3) in differentiated neurons was all suppressed by PPV-6, along with mitigation of caspase-3 cleavage. Further studies revealed that PPV-6 inhibited Aβ25-35 induction of SHH; indeed, PPV-6 was capable of suppressing neuronal CCR and apoptosis triggered by the exogenous N-terminal fragment of sonic hedgehog (SHH-N). Our findings demonstrated that, in the fully differentiated neurons, PPV-6 exerts protective actions against Aβ neurotoxicity via the downregulation of SHH to suppress neuronal CCR and apoptosis.

## 1. Introduction

Alzheimer’s disease (AD) is the major cause of dementia in the elderly with deteriorated cognitive functions accompanied by progressively impaired memory [1]. One prominent neuropathological hallmark of AD is senile plaques composed of amyloid-beta peptides (Aβs) of 39–43 amino acids, which are derived from proteolytic processing of amyloid precursor protein (APP) [2]. Among these Aβs, Aβ1-40 is the most abundant species, but Aβ1-42 with two additional hydrophobic amino acids tends to form insoluble aggregates that are pathologically more relevant [3]. Another shorter peptide fragment, Aβ25-35, is also capable of inducing apoptosis in primary cortical neurons [4]. Both intracellular and extracellular Aβs, including monomeric, oligomeric, and fibrillar forms, are considered to possess neurotoxicity [5,6]. Indeed, the “Amyloid Cascade Hypothesis” proposed that Aβ is responsible for activating all the detrimental downstream events of AD [1]. The molecular mechanisms underlying Aβ neurotoxicity include the heightened production of hydrogen peroxide [7] that may trigger oxidative stress [4], damages to mitochondria causing bioenergetic failure [8], neuroinflammation [9,10], and excitotoxicity [11], together causing synaptic degeneration [12] with resultant ultimate neuronal demise. Additionally, both Aβ1-42 and Aβ25-35 have been shown to modulate phospholipid turnover in microvessel pericytes and, together with endothelial cells, cause vascular damage during the course of amyloid accumulation [13,14].

In addition to the above-mentioned neurotoxicity of Aβs, neuronal cell cycle re-entry (CCR) has emerged as another pathogenic mechanism [15]. To complete cell division, proliferating cells undergo a series of tightly controlled steps, namely a cell cycle, that is composed of four different phases including the first gap phase (G1), the DNA-Synthesis phase (S), the second gap phase (G2), and the mitotic phase (M) [16]. Cyclins (A, B, D, E) and cyclin-dependent kinases (CDKs) are the two key regulators offering the stringent control of cell cycle progression [17]. Unlike most cell types, however, neurons are thought to lose their capability of proliferation following full differentiation, remaining at a quiescent state (G0 phase) in the adult nervous system [18]. Upon exposure to Aβs, however, the cell cycle program may be aberrantly reactivated in the differentiated neurons, resulting in cell death [19]. Clinical evidence suggested the potential involvements of CCR in neuronal loss observed in AD brains [20]. In AD patients, the re-expression of several protein markers for cell cycle progression suggested that neurons exit from G0; these markers include cyclins B, D, and E [21,22], proliferating cell nuclear antigen (PCNA), as well as Ki67 [18]. Previously we have reported that Aβs, including both aggregated Aβ25-35 and oligomeric Aβ1-42, can induce expression of sonic hedgehog (SHH), a morphogen pivotal to the development of embryonic nervous systems, in primary rat cortical neurons; the heightened expression of SHH is also observed in the cortical and hippocampal tissues of aged (up to 12-month-old) APPswe/PS1dE9 transgenic mouse brains [23]. Further, Aβ-induced SHH contributes to neuronal CCR and apoptosis; indeed, the N-terminal segment of SHH (SHH-N) capable of mimicking its biological action is sufficient to augment the expression of cell cycle markers in neurons, irrespective of Aβ [24]. Conceivably, the blockade of CCR in the fully differentiated neurons is expected to attenuate the extents of neuronal death as a result of Aβ exposure.

The use of naturally derived polysaccharides for intervention against AD or attenuation of Aβ toxicity has become more popular because of their multi-target beneficial effects and lower toxicity [25]. *Basella* species (spp.), also known as creeping spinach, Malabar spinach, or buffalo spinach, are perennial vines in the *Basellaceae* family. Several parts of *Basella* spp., including their leaves and stems, have been routinely consumed as vegetables on the dining table. Two common species, *Basella alba* with a green stem and leaves as well as *Basella rubra* with a reddish-purple stem and leaves, are found in tropical and subtropical areas. In traditional Chinese and Indian medicine, *Basella alba* has been used to treat various disorders [26]. Partial purification of *Basella* mucilage demonstrated that it contained polysaccharide mixtures [27] and glucan that can be separated by using starch iodine complex [28]. From the research literature, *Basella alba* has been shown to have beneficial actions against hypercholesterolemia and atherosclerosis [29]. Gavage feeding of the aqueous leaf extracts from *Basella alba* also offered significant hepatoprotection against paracetamol-induced hepatotoxicity in albino rats [30]. Despite these aforementioned reports, however, whether polysaccharides derived from *Basella alba* may carry beneficial effects against neurodegeneration has never been reported. In this study, we hypothesized that PPV-6, the polysaccharides prepared from the perennial vine *Basella alba*, may exert neuroprotective effects against Aβ toxicity. Using the fully differentiated primary rat cortical neurons as the experimental model, we tested whether PPV-6 may protect neurons from Aβ-mediated CCR and investigated the underlying mechanisms involving downregulation of SHH.

## 2. Results

### 2.1. Characteristics of PPV-6

Prior to examining its potential neuroprotective effects, we first determined different types of molecular weights of PPV-6; these include the number-average molecular weight (Mn), the peak molecular weight (Mp), the weight-average molecular weight (Mw), the Z-average molecular weight (Mz), and the polydispersity index (Mw/Mn) as shown below in Table 1.

We also characterized the monosaccharide composition of PPV-6 as shown below in Table 2. The results indicated that PPV-6 was mainly composed of glucose (Glc), galactose (Gal), and arabinose (Ara); in addition, PPV-6 also contained minor amounts of rhamnose (Rha), galacturonic acid (Gal-UA), and glucuronic acid (Glc-UA). Other monosaccharides like fucose (Fuc), xylose (Xyl), mannose (Man), fructose (Fru), ribose (Rib), guluronic acid (Gul-UA), and mannuronic acid (Man-UA) were either undetectable or not present in PPV-6.

### 2.2. PPV-6 Protects Primary Cortical Neurons against Aβ25-35 Toxicity

**Figure 1:**
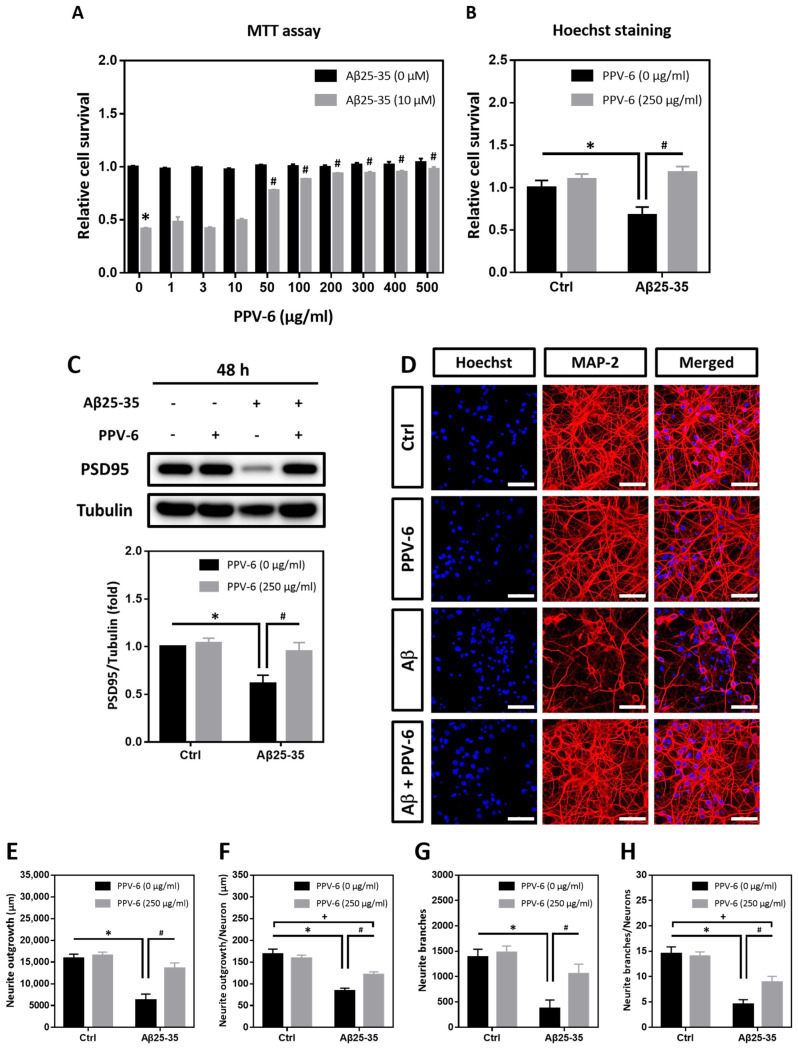
PPV-6 protects cortical neurons against Aβ25-35 toxicity with restoration of neuronal structure. (**A**) Primary cortical neurons were exposed to Aβ25-35 (10 μM) with or without PPV-6 at indicated concentrations for 48 h before MTT assay. Mean ± SEM from N = 4. * denotes *p* < 0.05 compared with control groups without Aβ25-35 treatment; # denotes *p* < 0.05 compared with Aβ25-35 groups without PPV-6 treatment. (**B**,**C**) Cortical neurons were exposed to Aβ25-35 (10 μM) with or without PPV-6 (250 μg/mL) for 48 h before Hoechst staining (**B**) or western blotting to detect expression of PSD-95 (**C**). Mean ± SEM from N = 3 in (**B**) and N = 5 in (**C**). * and # denote *p* < 0.05. (**D**–**H**) Cortical neurons were treated with or without Aβ25-35 (10 μM) or PPV-6 (250 μg/mL) for 48 h before immunostaining with the MAP-2 antibody (*red*) to label mature neurons; Hoechst 33258 (*blue*) served as the counterstaining. Scale bar = 50 μm. Quantitative analyses of total neurite length (in μm), mean neurite length (in μm) per neuron, total numbers of the neurite branch, and mean numbers of the neurite branch per neuron are shown respectively in (**E**–**H**). Mean ± SEM from N = 5. All data were analyzed by means of a one-way analysis of variance (ANOVA) followed by a *post-hoc* Tukey test. *, #, and + all denote *p* < 0.05.

MTT assay and Hoechst staining were performed to determine whether PPV-6 may confer resistance to differentiated cortical neurons against Aβ25-35 toxicity. As shown in Figure 1A, PPV-6 (50–500 μg/mL) dose-dependently increased the cell viability of cortical cultures exposed to Aβ25-35, reaching maximal efficacy at above 200 μg/mL. Hoechst staining indicated that PPV-6 at 250 μg/mL showed consistent protective effects (Figure 1B). Western blotting also demonstrated that PPV-6 recovered expression of the post-synaptic marker protein postsynaptic density-95 (PSD-95), which was downregulated by Aβ25-35 (Figure 1C). Therefore, the dosages of 200–250 μg/mL were selected for all the subsequent experiments. Besides biochemical assays, immunocytochemistry revealed that PPV-6 restored Aβ25-35-induced morphological damages in differentiated cortical neurons (Figure 1D). Quantitative analyses indicated that PPV-6 restored both total neurite lengths (Figure 1E) and average neurite length in each neuron (Figure 1F); PPV-6 also increased the neurite branches in each vision field (Figure 1G) and the average neurite branches of each neuron (Figure 1H). Together, these results indicated that PPV-6 can ameliorate Aβ25-35-mediated neurotoxicity in primary cortical cultures. 

### 2.3. PPV-6 Restores Neuronal Mitochondrial Function Compromised by Aβ25-35

**Figure 2:**
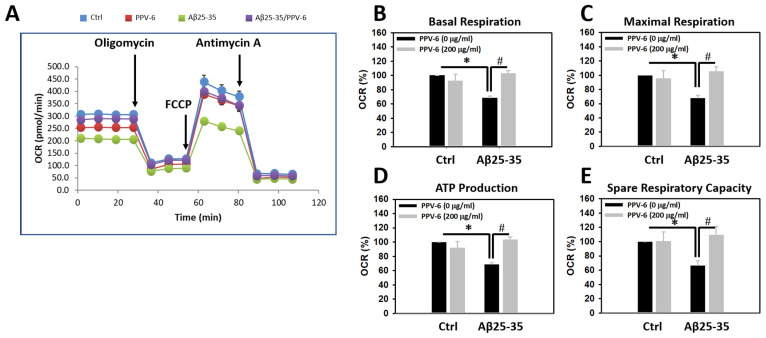
PPV-6 restores mitochondrial bioenergetic function impaired by Aβ25-35. Primary cortical cultures were exposed to Aβ25-35 (10 μM) with or without PPV-6 (200 μg/mL) for 24 h. One representative measurement of OCR is shown in (**A**); various indices for mitochondrial function, including basal respiration, maximal respiration, ATP production, and spare respiratory capacity are respectively shown in (**B**–**E**). Mean ± SEM from N = 4–5. Data were analyzed by means of a one-way ANOVA followed by a *post-hoc* Tukey test. * and # denote *p* < 0.05.

To test whether Aβ may trigger mitochondrial dysfunction in cultured neurons and, if so, whether PPV-6 is capable of reversing this detrimental effect of Aβ, mitochondrial bioenergetics were determined. Figure 2A demonstrates a representative plot showing real-time changes of oxygen consumption rate (OCR), as an index for mitochondrial function, following the addition of each mitochondrial inhibitor. Quantitative analyses revealed that the OCRs of basal respiration were significantly lower in the Aβ25-35-treated cultures, which were fully recovered by co-treatment with PPV-6 (Figure 2B). Similar results were obtained when the OCRs representing the maximal respiration rate (Figure 2C), ATP production (Figure 2D), and spare respiratory capacity (Figure 2E) were quantitatively determined. Together, the results shown in Figure 1 and Figure 2 collectively indicate that PPV-6 is capable of suppressing Aβ25-35-induced neurotoxicity with restoration of neuronal structure and improvements in mitochondrial bioenergetic functions.

### 2.4. PPV-6 Attenuates Cell Cycle Progression Induced by Aβ25-35

**Figure 3:**
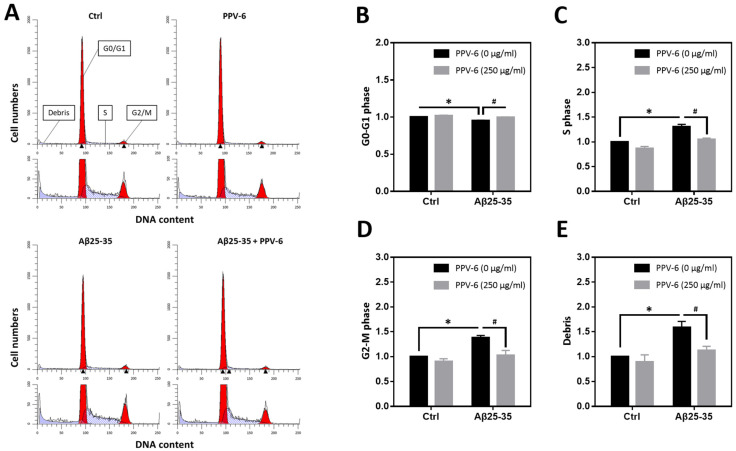
PPV-6 attenuates neuronal cell cycle progression induced by Aβ25-35 based on flow cytometry. Primary cortical neurons were exposed to Aβ25-35 (10 μM) with or without PPV-6 (250 μg/mL) for 16 h. This was followed by staining with propidium iodide (PI) and then flow cytometry to detect cellular DNA contents. Representative histograms showing the cell numbers in each phase, based on their relative DNA contents, are presented in (**A**). Quantitative analyses of the fold changes in cell numbers in the G0/G1 phases, S phase, and G2/M phases as well as cellular debris with fragmented DNA are respectively shown in (**B**–**E**). Mean ± SEM from N = 4. Data were analyzed by means of a one-way ANOVA followed by a *post-hoc* Tukey test. * and # denote *p* < 0.05.

We have previously reported that Aβ25-35 can trigger neuronal CCR and apoptosis [24,31]. Therefore, flow cytometry was conducted to determine cell cycle progression in the fully differentiated neurons at DIV-10 to test the PPV-6 effects on neuronal CCR. Representative histograms reveal that Aβ25-35 decreased the cell numbers in the G0/G1 phases while increasing those in the S- and G2/M phases; all were reversed by PPV-6 co-treatment (Figure 3A). Quantitative analysis confirmed these findings (Figure 3B–D). Consistent with a neurotoxic action, Aβ25-35 also increased the amount of cellular debris that was decreased by PPV-6 (Figure 3E).

### 2.5. PPV-6 Attenuates Aβ25-35- and Aβ1-42-Induced Expression of Cell Cycle Markers, Apoptosis, and BrdU Incorporation in Post-Mitotic Neurons

**Figure 4:**
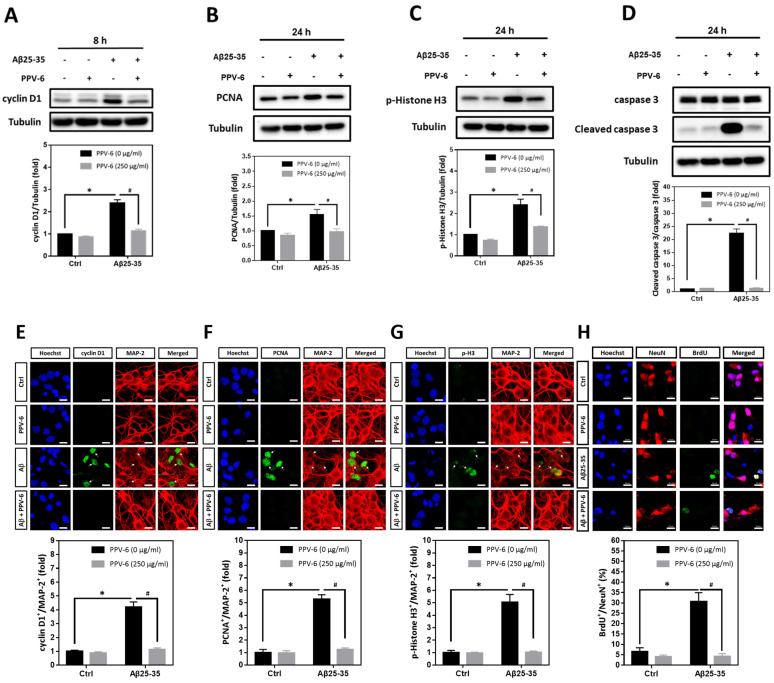
PPV-6 downregulates induction of the cell cycle/apoptosis markers and decreases the number of post-mitotic neurons incorporating BrdU upon Aβ25-35 exposure. (**A**–**D**) Primary cortical cultures were exposed to Aβ25-35 (10 μM) with or without PPV-6 (250 μg/mL) for 8 h before detection of cyclin D1 (**A**) or 24 h before detection of PCNA (**B**), p-Histone H3 (**C**), as well as pro- and cleaved caspase-3 (**D**). (**E**–**G**) Cortical neurons treated with Aβ25-35 (10 μM), PPV-6 (250 μg/mL), or both for 24 h were subjected to immunostaining with antibodies against various cell cycle markers (*green*), including cyclin D1 (**E**), PCNA (**F**), and p-Histone H3 (**G**); a MAP-2 (*red*) antibody stained the mature neurons. (**H**) The cultures were stained with the antibodies against BrdU (*green*) and neuronal marker NeuN (*red*); Hoechst 33258 (*blue*) served as counterstaining. White arrows denote the mature neurons expressing cell cycle markers or incorporating BrdU. Scale bar = 10 μm. Mean ± SEM from N = 3 in (**A**–**D**), N = 6 in (**E**–**G**), and N = 3 in (**H**). Data were analyzed by means of a one-way ANOVA followed by a *post-hoc* Tukey test. * and # denote *p* < 0.05.

Using cyclin D1, PCNA, and histone H3 phosphorylated at Ser-10 (p-Histone H3) as the respective marker for the G1 phase, S phase, and G2/M phases, we found that Aβ25-35-induced cyclin D1 was downregulated by PPV-6 (Figure 4A); Aβ25-35 also enhanced expression of PCNA (Figure 4B) and p-Histone H3 (Figure 4C) that were both suppressed by PPV-6. Most importantly, caspase-3 cleavage triggered by Aβ25-35 was completely blocked by PPV-6 (Figure 4D). Immunocytochemistry consistently indicated that Aβ25-35-induced cyclin-D1/MAP-2 co-localization was attenuated by PPV-6 (Figure 4E). PPV-6 also completely suppressed the Aβ25-35-induced increases in the number of PCNA^+^/MAP-2^+^ (Figure 4F) and p-Histone H3^+^/MAP-2^+^ (Figure 4G) neurons. BrdU incorporation coupled with immunocytochemistry may reveal de novo DNA synthesis during the S phase in the differentiated neurons. The results shown in Figure 4H clearly revealed that Aβ25-35 increased the number of BrdU^+^/NeuN^+^ cells that were completely abolished by PPV-6 co-treatment. Together, the results shown in Figure 3 and Figure 4 firmly established the notion that Aβ25-35 triggered CCR with resultant apoptosis in the differentiated cortical neurons, whereas PPV-6 effectively reversed these Aβ25-35 effects.

**Figure 5:**
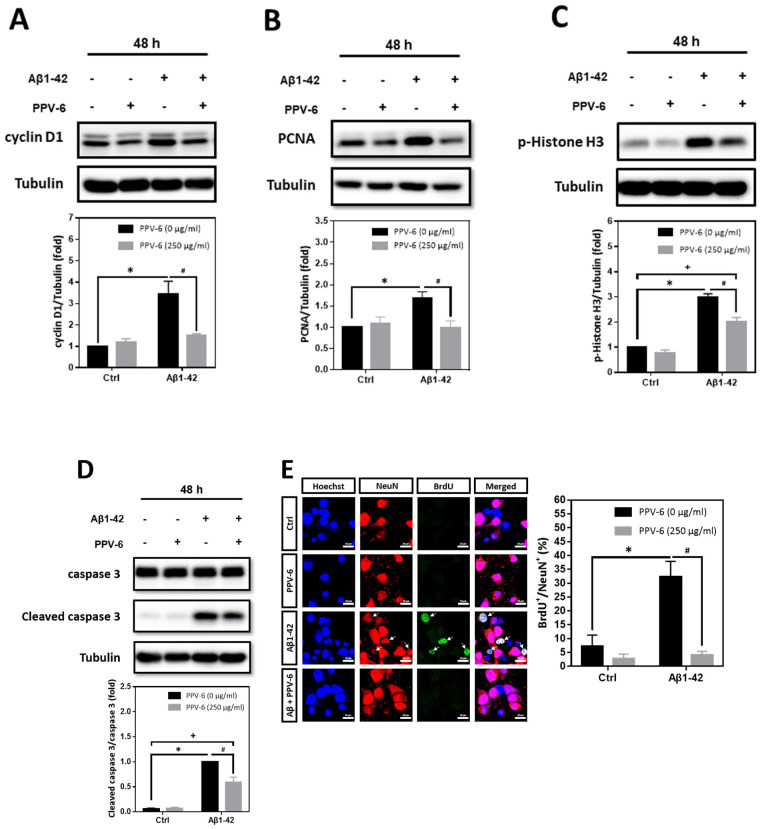
PPV-6 downregulates expression of the cell cycle/apoptosis markers as well as BrdU incorporation induced by Aβ1-42. (**A**–**D**) Primary cortical cultures were exposed to Aβ1-42 (5 μM), PPV-6 (250 μg/mL), or both for 48 h before detection of cyclin D1 (**A**), PCNA (**B**), p-Histone H3 (**C**), as well as pro- and cleaved caspase-3 (**D**). (**E**) The cultures were stained with antibodies against BrdU (*green*) and NeuN (*red*); Hoechst 33258 (*blue*) served as counterstaining. White arrows denote mature neurons with BrdU incorporation. Scale bar = 10 μm. Mean ± SEM from N = 3. Data were analyzed by means of a one-way ANOVA followed by a *post-hoc* Tukey test. *, #, and + all denote *p* < 0.05.

In addition to Aβ25-35, we also tested whether PPV-6 may carry similar protective effects against Aβ1-42. Western blotting showed that Aβ1-42-induced expression of cell cycle markers including cyclin D1 (Figure 5A), PCNA (Figure 5B), and p-Histone H3 (Figure 5C) were all attenuated by PPV-6. Aβ1-42-induced caspase-3 cleavage was also partially, but significantly, diminished by PPV-6 (Figure 5D). Moreover, Figure 5E revealed that Aβ1-42 increased the number of BrdU^+^/NeuN^+^ cells, which were completely abolished by PPV-6 co-treatment.

### 2.6. PPV-6 Blocks Aβ25-35-Induced Neuronal CCR in a Post-Treatment Paradigm

**Figure 6:**
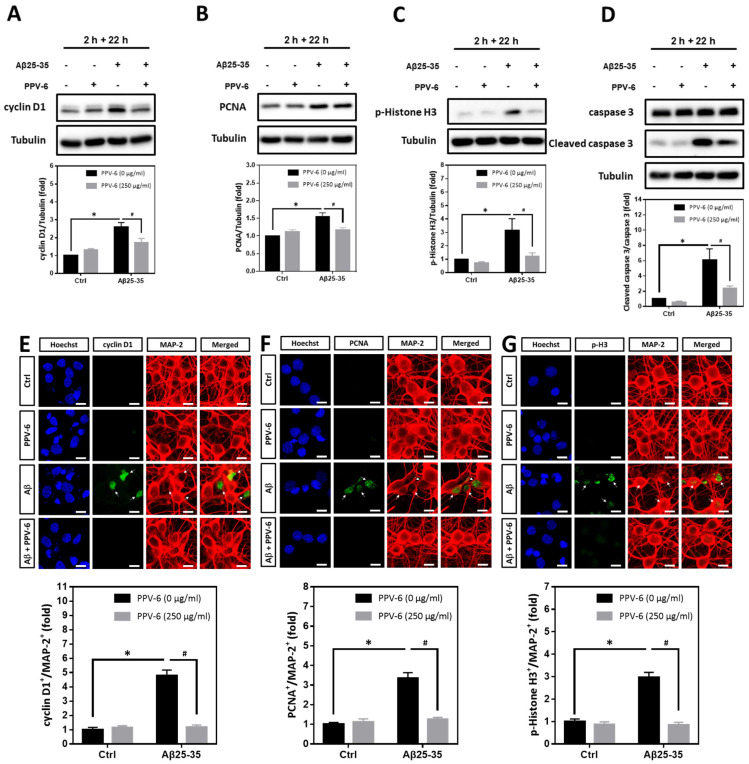
PPV-6 post-treatment downregulates expression of the cell cycle/apoptosis markers induced by Aβ25-35. (**A**–**D**) Primary cortical cultures were exposed to Aβ25-35 (10 μM) for 2 h; this was followed by treatment with PPV-6 (250 μg/mL) for additional 22 h in the absence of Aβ25-35 before detection of cyclin D1 (**A**), PCNA (**B**), p-Histone H3 (**C**), as well as both pro- and cleaved caspase-3 (**D**) through western blotting. (**E**–**G**) Similarly treated cortical cultures were subjected to immunostaining with antibodies against various cell cycle markers (*green*), including cyclin D1 (**E**), PCNA (**F**), and p-Histone H3 (**G**). The MAP-2 antibody (*red*) labeled the mature neurons; Hoechst 33258 (*blue*) served as counterstaining. White arrows denote the mature neurons positively stained with cell cycle markers. Scale bar = 10 μm. Mean ± SEM from N = 4 in (**A**–**D**) and N = 6 in (**E**–**G**). Data were analyzed by means of a one-way ANOVA followed by a *post-hoc* Tukey test. * and # denote *p* < 0.05.

We have confirmed that PPV-6 abolishes the neurotoxicity of both Aβ25-35 and Aβ1-42 in a co-treatment paradigm. Under such a condition, PPV-6 may physically associate with Aβs to block its neurotoxicity or, alternatively, trigger specific neuroprotective pathways in the cortical neurons independent of Aβs. To address this issue, we therefore tested whether PPV-6 may still protect cortical neurons against Aβ25-35 in a post-treatment paradigm. Cell cycle markers were analyzed in the post-mitotic neurons treated with 10 μM Aβ25-35 for 2 h followed by post-treatment with 250 μg/mL PPV-6, in the absence of Aβ25-35, for an additional 22 h. The results showed that Aβ25-35-induced expression of cell cycle marker proteins including cyclin D1 (Figure 6A), PCNA (Figure 6B), and p-Histone H3 (Figure 6C) were all significantly reversed by PPV-6 post-treatment. Moreover, exposure of cortical cultures to Aβ25-35 for only 2 h still triggered caspase-3 cleavage, which was attenuated by PPV-6 post-treatment for 22 h (Figure 6D). The exposure of differentiated cortical neurons to Aβ25-35 for 2 h consistently significantly increased the number of cyclin D1^+^/MAP-2^+^ (Figure 6E), PCNA^+^/MAP-2^+^ (Figure 6F), and p-Histone H3^+^/MAP-2^+^ (Figure 6G) cells; all were decreased by PPV-6 post-treatment. These findings thus indicated that PPV-6 may suppress neuronal CCR and apoptosis induced by Aβ25-35 by directly affecting cortical neurons, possibly by triggering specific signaling pathways, independently of Aβ25-35 to result in the observed neuroprotection.

### 2.7. PPV-6 Inhibits Aβ25-35-Induced Expression of SHH and SHH-N-Induced Neuronal CCR 

**Figure 7:**
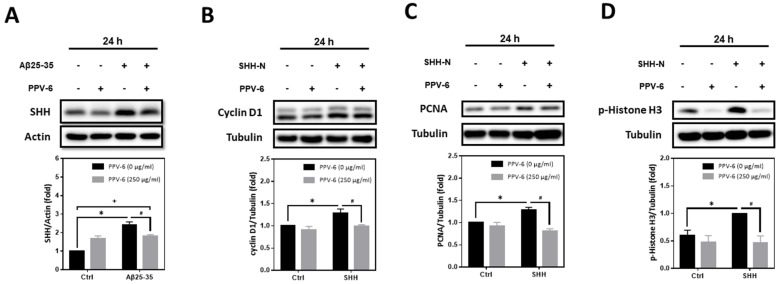
PPV-6 suppresses Aβ-induced SHH expression and blocks SHH-N-mediated neuronal CCR. (**A**) Primary cortical cultures were exposed to Aβ25-35 (10 μM) with or without PPV-6 (250 μg/mL) for 24 h before detection of SHH by means of western blotting. (**B**–**D**) Primary cortical cultures were exposed to SHH-N (300 ng/mL) with or without PPV-6 (250 μg/mL) for 24 h before detection of various cell cycle markers, including cyclin D1 (**B**), PCNA (**C**), and p-Histone H3 (**D**). Mean ± SEM from N = 4 in (**A**), N = 3 in (**B**), N = 3 in (**C**), and N = 5 in (**D**). Data were analyzed by means of a one-way ANOVA followed by a *post-hoc* Tukey test. *, #, and + all denote *p* < 0.05.

Previously, we have demonstrated that Aβs may induce the expression of SHH via sequential induction of the inhibitor of differentiation-1 (Id1) and hypoxia-inducible factor-1 (HIF-1), thereby establishing a signaling cascade of “Aβ → Id1 → HIF-1 → SHH” in primary cortical neurons [23]. Further mechanistic investigation revealed that Id1, HIF-1, and SHH contribute to Aβ-induced neuronal CCR with resultant apoptosis; indeed, SHH-N and cobalt chloride (CoCl_2_)—the latter is known to stabilize HIF-1α [32]—are both sufficient to trigger neuronal CCR and apoptosis independent of Aβs [24,31]. In the present study, we therefore tested whether PPV-6-dependent neuroprotection is due to its inhibitory effects on Aβ-mediated SHH induction, thereby downregulating the subsequent neuronal CCR and apoptosis, or, alternatively, whether PPV-6 is capable of directly suppressing neuronal CCR independently of Aβ-induced SHH expression. The results shown in Figure 7A clearly indicated that PPV-6 did significantly suppress Aβ25-35-induced SHH expression, although only partial suppression was observed. These findings appeared to support the contention that PPV-6 suppresses neuronal CCR induced by Aβ by blocking SHH expression, at least in part. Interestingly, when PPV-6 was co-incubated with SHH-N that is sufficient to trigger neuronal CCR [24], we found that PPV-6 also inhibited SHH-N-mediated induction of cell cycle markers, including cyclin-D1 (Figure 7B), PCNA (Figure 7C), and p-Histone H3 (Figure 7D). These observations indicated that PPV-6, besides downregulation of Aβ-mediated SHH expression, is also capable of suppressing SHH-N-induced CCR independently of Aβs in the fully differentiated post-mitotic cortical neurons. 

**Figure 8:**
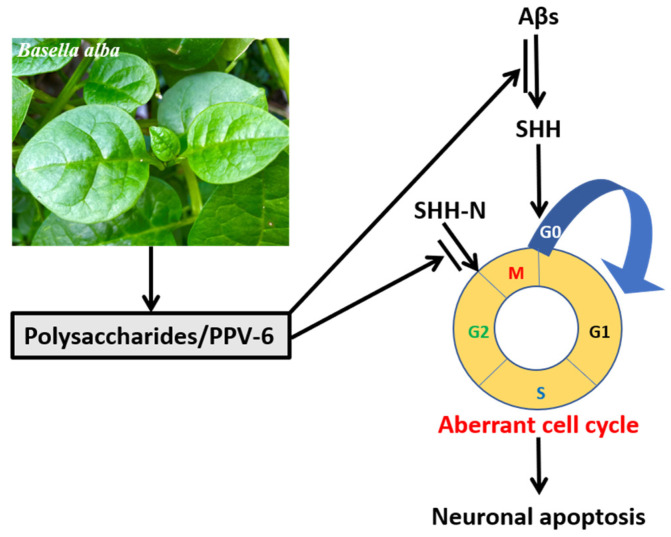
PPV-6 exerts neuroprotective effects in part via suppression of Aβ-induced SHH expression and subsequent activation of CCR followed by apoptosis in the fully differentiated post-mitotic neurons. Further, PPV-6 is also capable of directly inhibiting neuronal CCR triggered by exogenous SHH-N in cortical neurons.

Overall, the results shown in the present study demonstrated that, in the fully differentiated neurons, PPV-6 exerts protective actions against Aβ neurotoxicity via downregulation of SHH to suppress neuronal CCR and apoptosis; further, PPV-6 may also suppress neuronal CCR triggered by SHH-N independently of Aβ. A simple scheme depicting our results in this work is shown in Figure 8.

## 3. Discussion

One of the most important variables affecting the biological activity of polysaccharides is their structural features, such as monosaccharide composition, molecular weight, the extent of branching, and linkage structure; the complexity of polysaccharide structure makes the studies of its structure–activity relationship very difficult [25]. In the present study, we reported that glucose, galactose, and arabinose are the major monosaccharide components in PPV-6, accounting for approximately 95.4% of total polysaccharides; minor amounts of rhamnose, galacturonic acid, and glucuronic acid are also present. These findings are similar to the findings in one recent study that polysaccharides of *Basella alba* prepared by ethanol precipitation mainly contained D-galactose and L-arabinose, with minor amounts of rhamnose and galacturonic acid [33]. High arabinose and galactose ratios might be an index for the presence of arabinogalactans [34]. A homogeneous neutral polysaccharide designated LBP1A1-1 with an average molecular weight of 45.0 kDa was purified from fruits of *Lycium barbarum* L.; interestingly, LBP1A1-1 was also an arabinogalactan that inhibited the aggregation of Aβ1-42 in vitro [35]. Besides arabinogalactan, a polysaccharide composed of only glucose LJW0F2, which was a neutral glucan purified from the flowers of *L. japonica* Thunb, also directly inhibited Aβ1-42 aggregation and attenuated its neurotoxicity in vitro [36]. These previous findings suggest the possibility that neutral polysaccharides, such as arabinogalactans and glucan, may block the aggregation of Aβs to exert their anti-AD effects. In a different study, however, another neutral glucan CCP with an average molecular weight of 3.96 kDa purified from *Coptis chinensis* protected PC12 cells against Aβ25-35 toxicity by inhibiting c-Jun N-terminal kinase (JNK)-mediated pro-apoptotic pathways; because in this work PC12 cells were treated with CCP first followed by challenges with Aβ25-35 in a pretreatment paradigm, the possibility that CCP directly interfered with the aggregation of Aβ25-35 to neutralize its toxicity can be excluded [37]. This work also implies that neutral glucan CCP is capable of directly triggering signal transduction pathways in vitro for neuroprotection without affecting Aβ aggregation. In our study, the molecular structures of PPV-6 have not been characterized yet. Nevertheless, besides the co-treatment paradigm (Figure 1), PPV-6 also protected cortical neurons against Aβ25-35 toxicity in the post-treatment paradigms (Figure 6), wherein the cortical neurons were challenged with Aβ25-35 first, followed by rescue with PPV-6 in the absence of Aβ25-35 (Figure 6). These findings appeared to exclude the possibility that PPV-6 protects cortical neurons simply by blocking aggregation of Aβ25-35, at least in the post-treatment paradigm. Regardless of its molecular structure, PPV-6 is capable of directly affecting the cortical neurons and downregulating the Aβ-induced SHH expression with attenuation of neuronal CCR and apoptosis. The structural components responsible for this protective effect require further characterization of PPV-6 in detail.

Potential antioxidative effects of *Basella alba* in various disease models have previously been proposed. For example, the methanol extract of *Basella alba* leaves lessens the extent of free radical production due to stress induced by restraint and forced swimming in rats [38]. The fruit pulp extracts of *Basella alba* also ameliorate the testicular histopathology induced by carbon tetrachloride (CCl_4_), which is known to cause oxidative stress, in albino mice [39]. The extracts of leaves and seeds derived from *Basella alba* effectively scavenge reactive oxygen species (ROS) with antioxidant activity in Ehrlich ascites carcinoma (EAC) cells [40]. In male Wistar rats intraperitoneally injected with streptozotocin to induce diabetes mellitus, gavage feedings with the aqueous extracts of *Basella alba* leaves also enhance the enzymatic activity of superoxide dismutase (SOD) and the reducing ability of serum ferric iron in the circulating blood, thereby indirectly alleviating oxidative stress in the gonadal tissues [41]. Indeed, one recent study has explored the nutritional components and antioxidant capacity among different organs, such as green fruits, black fruits, leaves, and stems, derived from eight typical cultivars of *Basella alba* [42]. Although we have not directly examined its potential antioxidative effects in neurons, PPV-6 did restore the mitochondrial bioenergetic capabilities that were compromised by Aβ25-35 (Figure 2). Given that mitochondrion is the major organelle producing ROS, the recovery of mitochondrial functions may imply mitigation of oxidative stress in the cortical neurons exposed to Aβs.

In the present work, we reported that PPV-6 may block neuronal CCR with anti-apoptotic effects by suppressing Aβ-induced expression of cell cycle markers, such as cyclin-D1 (Figure 4A, Figure 5A and Figure 6A) and pRb phosphorylation (Wu and Yang, unpublished observations), and caspase-3 cleavage (Figure 4D, Figure 5D and Figure 6D) in post-mitotic neurons. Consistently, methanol extracts of *Basella alba* leaves induced cell cycle arrest at the G0/G1 phase in colon cancer cell lines with repression of cyclin D1 expression and pRb phosphorylation; in the same study, BaME also dose-dependently suppressed EGFR, pERK, and pAkt pathways [43]. In our work, we reported that PPV-6 suppressed Aβ-induced cell cycle markers such as cyclin-D1 in the post-mitotic neurons by downregulating SHH, which is also considered a mitogen capable of sustaining cell cycle progression [44]; however, whether EGFR, pERK, and pAkt were also affected by PPV-6 in the Aβ-treated neurons requires additional investigation. Despite its actions in suppressing cell cycle progression, however, the anti-apoptotic effects of PPV-6 shown in the present study appeared to contradict the earlier report demonstrating that leaf extracts from *Basella alba* trigger apoptosis by regulating expression of several related genes, including caspase-3, in EAC cells [45]. The rationales behind such discrepancies remain to be determined, but one major reason may be the experimental models used in these different studies. This suggests that, although naturally derived polysaccharides may have regulatory effects on cell cycle progression, the underlying mechanisms in post-mitotic neurons may be different compared to those in proliferating cancer cells.

One interesting finding presented in this work is that PPV-6 possesses the capabilities of suppressing Aβ-induced expression of SHH (Figure 7A), but the detailed underlying mechanisms still remain unknown. Three potential mechanisms may be speculated. First, we have previously reported that Aβ-induced SHH expression depends on the inhibitor of differentiation-1 (Id1) and hypoxia-inducible factor-1 (HIF-1), thus establishing the signaling cascade of “Aβ → Id1 → HIF-1 → SHH” in rat cortical neurons [23]. Whether PPV-6 may affect the Id1/HIF-1 pathway to suppress the Aβ-induced SHH expression requires further investigation. Second, our preliminary results indicated that Aβ25-35 may also enhance the binding of the p50 subunit of the nuclear factor-kappaB (NF-κB) to the *Shh* gene promoter, thereby contributing to its expression (Hsieh and Yang, unpublished observations). Interestingly, the dehydrated *Basella alba* colorant powder (BACP) carries anti-inflammatory activity and blocks NF-κB activation in lipopolysaccharide (LPS)-treated RAW 264.7 macrophages [46]. It is therefore intriguing to speculate on the possibility that PPV-6 may also inhibit Aβ-induced NF-κB activation to suppress the expression of SHH. Finally, we have reported that SHH mediates the neuroprotective effects of brain-derived neurotrophic factor (BDNF) against mitochondrial inhibitor 3-nitropropionic acid (3-NP) in cortical neurons [47]; further, BDNF-dependent SHH expression and 3-NP resistance require the prior induction of erythropoietin (EPO), thus establishing a signaling cascade of “BDNF → EPO → SHH → 3-NP resistance” in rat cortical neurons [48]. Given that PPV-6 fully recovered Aβ25-35-induced mitochondrial dysfunctions (Figure 2), it is interesting to speculate whether PPV-6 may modulate the BDNF receptor tropomyosin-related kinase (TrkB) or EPO receptor to affect expression of SHH. These possible mechanisms underlying PPV-6-dependent suppression of SHH induction by Aβ all require further investigation. Apart from suppression of SHH expression to attenuate neuronal CCR induced by Aβ, PPV-6 also blocked exogenous SHH-N-induced neuronal CCR independent of Aβ (Figure 7B–D). Since Aβ is not the only mediator capable of reactivating the cell cycle causing apoptosis in the post-mitotic neurons, these results suggest that PPV-6 may also carry beneficial effects in other disease models wherein CCR is activated and contributes to neuronal demise. For example, 1-methyl-4-phenylpyridinium (MPP^+^), commonly used in studies on Parkinson’s disease, also triggers neuronal CCR and cell death in rat cerebellar granule neurons (CGNs) [49]. The possibility of the broader application of PPV-6 in the contexts of other neurodegenerative diseases can be speculated on.

Besides potential anti-oxidative actions and anti-apoptotic actions, other beneficial effects of PPV-6 may deserve further exploration. As mentioned above, anti-inflammatory action of the dehydrated BACP has been characterized in LPS-treated macrophages capable of downregulating various pro-inflammatory mediators along with the blockade of NF-κB activation [46]. At molecular level, one recent molecular docking study was performed to observe the pharmaceutical impact of the fruit extracts derived from *Basella alba* on the anti-inflammatory cyclooxygenase-2 (COX-2) enzyme [50]. Moreover, polysaccharides from *Lycium barbarum* promote hippocampal neurogenesis with improvements in the impaired synaptic plasticity in an AD transgenic mouse model [51]. Regardless of the detailed underlying mechanisms, the beneficial roles of *Basella* species in neurodegenerative diseases like AD or other brain disorders have never been reported. The present work, to our best knowledge, is the first study associating polysaccharides extracted from *Basella alba* with neuroprotective effects against Aβ toxicity.

Despite our positive findings shown in the present study, certain limitations in this research should be considered. First, since the cortical neurons differentiated in vitro were used in the present study, whether the polysaccharides extracted from the medicinal plants may gain direct access to the neurons in vivo remains a critical issue. In particular, there is still debate about whether the polysaccharides can enter the blood and further penetrate the blood–brain barrier (BBB) into the central nervous system in vivo. One earlier study was therefore initiated to search for BBB-permeable natural polysaccharides with neuroprotective efficacies; the authors reported that a 4.7-kDa polysaccharide derived from low acyl gellan gum (LA-GAGR), termed midi-GAGR, demonstrates relatively good BBB permeability and neurotrophic effects in the brains of AD transgenic mice [52]. This report suggests that at least certain types of polysaccharides may be capable of crossing the BBB, although the molecular weight of PPV-6 at 26.26 kDa appears much larger than the reported 4.7 kDa [52]. In order to address this issue, gavage feeding of PPV-6 into the transgenic mouse model, such as APPswePS1dE9 mice, followed by behavioral tests and the examination of pathological outcomes like oxidative stress and neuroinflammation may allow for direct assessments of the beneficial effects exerted by PPV-6 in vivo. It should also be noted that, despite the observed neuroprotective effects of PPV-6 against Aβs in vitro, the possibility that PPV-6 carries other beneficial actions in AD via alternative pathways cannot be overlooked. For example, microbiota dysbiosis may boost various pathogenic mechanisms of AD like immunosenescence, oxidative stress, and neuroinflammation [53]. Gut microbiota can also affect various critical pathways, such as APP processing, to influence the incidence of AD by the gut–brain axis [54]. Recently, yeast β-glucan has been shown to ameliorate cognitive deficits by regulating gut microbiota and metabolites in the Aβ1-42-induced AD-like mouse model [55]. Thus, even under the scenario that PPV-6 has only limited BBB permeability in vivo, it may still carry possible therapeutic effects for AD. These potential beneficial functions of PPV-6 await further exploration in the physiologically more relevant experimental model system, such as AD transgenic mice. The second limitation is that the active ingredients in PPV-6 with neuroprotective action remain to be fully characterized. Since polysaccharides extracted from plants are a mixture of macromolecules, it is difficult to analyze and precisely pinpoint the active ingredients. Our results shown in Table 2 clearly revealed the monosaccharide composition of PPV-6. However, at present we cannot fully exclude the possibility that other components, such as small-molecule phytochemicals or other unknown substances, along with the polysaccharides in PPV-6, also contributed to the observed neuroprotective effects against Aβs. Nevertheless, it should be noted that PPV-6 used in the present work was acquired by extraction with high-temperature (90 °C for 2 h) water followed by precipitation with 95% ethanol with subsequent freeze-drying into powder. These manipulations are expected to substantially decrease, if not completely eliminate, the contents of small-molecule phytochemicals. Thus, the biologically active components in PPV-6 with neuroprotective efficacy still await further identification. The third limitation is that, in this work, primary neuronal cultures were challenged with pre-aggregated Aβs, including Aβ25-35 and Aβ1-42, to examine the potential neuroprotective effects of PPV-6. However, the interpretation of results derived from this in vitro model requires additional caution. For example, the application of pre-aggregated Aβs may trigger downstream pathogenic pathways like oxidative stress and apoptosis, but this experimental system cannot reproduce the pathological processes before the formation of Aβs, namely APP processing and aggregation. In addition, a short-term exposure (24–48 h) of Aβs may not fully recapitulate the physiological and pathological situations of AD caused by chronic expression of mutant APP or presenilin (PS), which takes decades. Further, neural progenitor cells derived from E-18 fetal cortices are subjected to in vitro differentiation, which may not be ideal for studies of the age-dependent neurodegenerative processes occurring in AD. Finally, two-dimensional monolayer neuronal culture cannot fully recapitulate the pathophysiological conditions of three-dimensional (3D) structures in the brain. Recently, a 3D model using genetically engineered human neural stem cells that overexpress APP and PS1 with fAD mutations was reported [56,57]. In this improved in vitro cell model, aggregation of Aβs was observed in a 3D Matrigel culture system after approximately 6 weeks of differentiation. We have already established such a “humanized” 3D AD model (Hsieh and Yang, unpublished observations), which can be a valuable in vitro tool to test the neuroprotective efficacy of PPV-6 in the near future.

In the present study, we reported that the crude polysaccharides extracted from *Basella alba* exhibit neuroprotective efficacies in the in vitro model of AD. Several future directions may be envisaged to further advance its therapeutic or preventive applications in AD or other neurodegenerative disorders. First, PPV-6 may be digested with α-amylase, amyloglucosidase, and protease for the removal of starch and proteins, thereby acquiring the more refined PPV-6. Such refined PPV-6 may be applied to the in vitro experimental system using Aβ-treated neuronal cultures or gavage feeding to AD transgenic mice for in vivo studies. Second, the refined polysaccharide from *Basella alba* can also be further fractionated through ion-exchange chromatography and gel filtration chromatography; each of the collected fractions may then be subjected to tests for their neuroprotective efficacy against Aβ toxicity in primary cortical neurons for the identification of their most effective ingredients before being used for gavage feeding into APPswePS1dE9 AD transgenic mice. Finally, prior to the translation of PPV-6 into clinical settings for AD or other neurodegenerative disorders, it is imperative to firmly establish its biological safety, despite the fact that it is derived from a commonly consumable vegetable. Various methods can be applied to demonstrate that the consumption of PPV-6 does not cause any concerns for toxicity in humans or animals. These include a mammalian erythrocyte micronucleus test to analyze the peripheral blood reticulocytes for the presence of micronuclei as an index for chromosomal aberration [58], an Ames test for mutagenicity [59], a 24 h acute toxicity test in rats by means of gavage feeding, and a 28 d sub-acute toxicity test in mice by means of gavage feeding. Once accomplished, these studies may pave the foundation for the clinical application of PPV-6 in clinical settings for AD or other neurodegenerative disorders.

In conclusion, our findings indicated that the neuroprotective mechanisms of PPV-6 involve, at least in part, suppression of SHH expression as well as neuronal CCR and subsequent apoptosis induced by Aβs. Polysaccharides derived from *Basella alba* may thus carry therapeutic potential for AD.

## 4. Materials and Methods

### 4.1. Plant Materials and Preparations of PPV-6

*Basella alba* was cultivated in Zhuangwei district, Yilan, Taiwan. The plant materials were identified as *Basella alba* by Mr. Wen-Hwa Lin, who is an Assistant Researcher at the Hualien District Agricultural Research and Extension Station (Council of Agriculture, Executive Yuan, Taiwan). The PPV-6 was prepared by Mynature Biotech Inc., Yilan, Taiwan, as described in detail below. Fresh aerial parts of *Basella alba* were minced and grounded followed by aqueous extraction for 2 h at 90 °C. After filtration to remove the debris, 95% ethanol (*w*/*w*) was added into the aqueous extract with a ratio of 3:1 at 4 °C for 24 h to precipitate the crude polysaccharides; this was followed by filtration to collect the pellet, and finally the pellet samples were freeze-dried into powder, or PPV-6, and stored at room temperature until use. Prior to experimentation, the PPV-6 was dissolved in sterile water to make a stock solution of 5 mg/mL; the solution was then autoclaved for 30 min followed by centrifugation at 16,000× *g* for 1 min to obtain the supernatant, with a final working concentration of PPV-6 at 200–250 μg/mL in culture medium, depending on the experimental conditions.

### 4.2. Determination of the Molecular Weights and Monosaccharide Compositions of PPV-6

PPV-6 were characterized according to the previously published methods [60,61,62] to determine the molecular weights and monosaccharide compositions. The detailed protocols were included in the “Supplementary Materials: Detailed Experimental Protocols”.

### 4.3. Preparations of Aβs, SHH-N, and Primary Cortical Neurons 

Aβ25-35 (Cat. No. A4559, Sigma-Aldrich, St. Louis, MO, USA) was prepared as a 2-mM stock solution in the autoclaved ddH_2_O and stored at −80 °C; aliquots of Aβ25-35 were incubated at 37 °C for 24 h to permit its aggregation into fibrils, with a final working concentration at 10 μM, before application to cortical neurons [24,31]. Aβ1-42 (Cat. No. A–1163–2, rPeptide Inc., Watkinsville, GA, USA), previously treated with 1,1,1,3,3,3-hexafluoro-2-propanol (HFIP) to give a highly monomeric starting material, was dissolved in 10% DMSO in phosphate-buffered saline (PBS) at 100 μM and stored at –80 °C. Prior to experimentation, the Aβ1-42 stock solution was incubated at 4 °C for 24 h to allow aggregation into oligomers. SHH-N (Cat. No. 461-SH, R&D Systems, Minneapolis, MN, USA) was dissolved in sterile PBS to make a stock solution of 50 μg/mL, with the final working concentration at 300 ng/mL in a culture medium. Neuron-enriched primary cortical cultures were derived from fetal brain cortices of Sprague–Dawley rats and used for between 7 and 10 days in vitro (DIV) as previously described [63]. The detailed protocols for primary cultures of fetal rat cortical neurons were included in the “Supplementary Materials: Detailed Experimental Protocols”.

### 4.4. Cell Survival Assays

The MTT (3-[4,5-dimethylthiazol-2-yl]-2,5-diphenyl-tetrazolium bromide; Cat. No. SI-M5655, Sigma-Aldrich) reduction assay measured the extent of cell viability because the surviving cells with functional mitochondria are capable of reducing the yellowish MTT solution into dark blue formazan precipitates with the maximal absorbance at 570 nm. Hoechst 33258 was used to stain the viable cells with normal nuclear morphology. Both assays were performed as described in our earlier publication [64] and the detailed protocols were included in the “Supplementary Materials: Detailed Experimental Protocols”.

### 4.5. Immunocytochemistry and Quantification of Neurite Lengths and Neurite Branches

Immunocytochemistry was performed as described in detail in our earlier publication [24]. The following rabbit antibodies were used for the cell cycle: cyclin D1 (1:100; Cat. No. ab134175, Abcam, London, UK), PCNA (1:100; Cat. No. ab92552, Abcam), and p-Histone H3 (1:100; Cat. No. ab32107, Abcam). Mouse monoclonal antibodies against MAP-2 (1:100; Cat. No. MAB378, CHEMICON International, Temecula, CA, USA) were used to stain the mature neurons. A rat antibody against 5-bromo-2′-deoxyuridine (BrdU; 1:200; Cat. No. 6326, Abcam) was used to label the cells undergoing de novo DNA synthesis; a rabbit antibody against the neuronal nuclear protein (NeuN; 1:100; Cat. No. ABN78, Sigma-Aldrich) labeled the nuclei of the mature neurons. A laser-scanning confocal microscope (Zeiss LSM700, Oberkochen, Germany) equipped with filter sets was used to observe the corresponding fluorescence signals. To determine the neuronal structure, the Neurite Outgrowth image analysis module in MetaMorph software (Version 7.7, Molecular Devices, LLC., San Jose, CA, USA) was used to identify and measure neurite outgrowth and nuclei. The software automatically calculates and reports measurements such as the neurite length and the number of neurite branches. The measurements were then exported for further analysis and visualization.

### 4.6. Mitochondrial Bioenergetics and Flow Cytometry

The OCR was measured by a XF24 Extracellular Flux Analyzer (Seahorse Bioscience, Billerica, MA, USA) to determine the mitochondrial bioenergetics according to our previously described protocols [65]. For flow cytometry to determine cell cycle progression, rat cortical cultures were digested with 0.5% trypsin and 7 mM EDTA at 37 °C for 7 min. After the addition of Dulbecco’s Modified Eagle Medium (DMEM; Cat. No. 12800-017, GIBCO/Thermo Scientific, Waltham, MA, USA), the cell suspension was centrifuged at 180× *g* for 5 min before fixation in cold 70% ethanol. For cell cycle analysis, fixed cells were subjected to two washes with PBS and digested with RNase (100 μg/mL) for 1 h at 37 °C before staining with propidium iodide (PI; 10 μg/mL; Cat. No. P4170, Sigma-Aldrich) for 30 min in the dark. For DNA content analysis, the single cell population was picked up based on the FSC-H-FSC-A scatter plot. The PI intensity of each cell in a single population was recorded and illustrated as a histogram to construct the DNA content distribution. DNA content was assessed by using a BD FACSCanto (BD Medical Technology, Franklin Lakes, NJ, USA) and cell cycle distribution was analyzed by using ModFit LT software (Version 5.0, Cytonome Verity, LLC., Bedford, MA, USA).

### 4.7. Western Blotting

Western blotting was carried out as described in our previously published paper [24]. Rabbit antibodies against caspase-3 (1:1000; Cat. No. 9662, Cell Signaling Technology, Danvers, MA, USA), a mouse antibody against α-tubulin (1:10,000; Cat. No. SI-T9026, Sigma-Aldrich), and a mouse antibody against β-actin (1:5000; Cat. No. MAB1501, Sigma-Aldrich) were all diluted in blocking buffer (5% nonfat dry milk in PBST buffer containing 0.05% Tween-20). Rabbit antibodies against cyclin D1 (1:1000; Cat. No. ab134175, Abcam), PCNA (1:1000; Cat. No. ab92552, Abcam), p-Histone H3 (1:1500; Cat. No. ab32107, Abcam), cleaved caspase-3 (1:1000; Cat. No. 9664, Cell Signaling Technology), and SHH (1:500; Cat. No. 2207, Cell Signaling Technology) were diluted in signal enhancer HIKARI solution 1 (Cat. No. NT08044-71R, Nacalai Tesque, Kyoto, Japan). Horseradish peroxidase (HRP)-conjugated anti-rabbit or anti-mouse secondary antibodies were applied in fresh blocking buffer at 1:5000 to detect the corresponding primary antibodies for caspase-3, cyclin D1, PCNA, p-Histone H3, cleaved caspase-3, and α-tubulin. α-tubulin or β-actin was included as an internal control for the equal loading of proteins in each lane. Immunoreactive signals were detected using ECL-Plus Western blotting detection reagents (Cat. No. FL0010-0125, Bionovas, Toronto, ON, Canada). The blots were analyzed under a Luminescence Imaging System Amersham Imager 600 (FUJIFILM, Tokyo, Japan) and signal intensity quantified using ImageJ software (Version 1.44p, National Institutes of Health, Bethesda, MD, USA).

### 4.8. Statistical Analysis

Statistical analyses were performed as described in our previous publications [24,31]. The results are expressed as mean ± SEM from the sample number (N). Data were analyzed by means of a one-way analysis of variance (ANOVA) followed by a *post-hoc* Tukey test. *p*-values less than 0.05 are considered statistically significant.

## Figures and Tables

**Table 1:** Estimated molecular weights (in kDa) of PPV-6; Mw/Mn indicates the polydispersity index.

Mn	Mp	Mw	Mz	Mw/Mn
26.26 ± 0.20	72.27 ± 0.03	134.14 ± 0.02	452.89 ± 0.03	5.11 ± 0.20

**Table 2:** Carbohydrate compositions (in mol %) of PPV-6; Fuc, Xyl, Man, Fru, Rib, Gul-UA, and Man-UA are non-detectable.

Glc	Gal	Ara	Rha	Gal-UA	Glc-UA
40.20 ± 0.27%	30.76 ± 0.41%	24.40 ± 0.09%	3.54 ± 0.04%	0.73 ± 0.18%	0.38 ± 0.01%

## Data Availability

All data generated or analyzed during this study are included in this published article.

## Supplementary Materials

The following supporting information can be downloaded at: https://www.mdpi.com/article/10.3390/ijms25137316/s1, Supplementary Materials: Detailed Experimental Protocols; PPV-6 and Abeta-Full Blots. References [66,67,68,69] are cited in the Supplementary Materials.
