# Supplementary material for: Polysaccharides from Basella alba Protect Post-Mitotic Neurons against Cell Cycle Re-Entry and Apoptosis Induced by the Amyloid-Beta Peptide by Blocking Sonic Hedgehog Expression
Source: Int J Mol Sci. 2024 Jul 3;25(13):7316. doi: 10.3390/ijms25137316 (PMC11242684; doi:10.3390/ijms25137316)
Supplement: Supplementary file 1 [file ijms-25-07316-s001.zip › ijms-3006021-supplementary.pdf]

File Name: Supplementary Materials - Detailed Experimental Protocols
Manuscript No.: ijms-3006021R-R1
Title: Polysaccharides from *Basella alba* protect post-mitotic neurons against cell cycle reentry and apoptosis induced by amyloid-beta peptide by blocking sonic hedgehog expression
Authors: Bo-Yu Hou, Ming-Hsuan Wu, Hui-Yu Hsu, Yi-Chun Lin, Ding-I Yang

Primary cultures of fetal rat cortical neurons

Cortical neurons were cultured from fetal brains of Sprague-Dawley rats. Briefly, cortices were dissected mechanically from fetal rat brains at embryonic day-18 (E-18) and then triturated through pipettes in MEM medium supplemented with 10% FBS. This was followed by filtering through 70-μm filter into an autoclaved tube and immediately centrifuged at 115× g for 10 min. The pellet containing cortical cells was resuspended in MEM medium. After gentle mixing, cells were plated at a density of approximately 1×10^6 cells/ml in the culture vessels previously coated with poly-D-lysine. After 4 h of incubation, the culture medium was replaced with Neurobasal medium supplemented with B27 (GIBCO/Invitrogen Corporation, Carlsbad, CA). The cultured cortical cells were maintained in a humidified incubator at 37°C with 5% CO_2 for at least 7 days to allow the regeneration of axons and dendrites. Experiments were conducted on neurons cultured between 7 and 10 days-*in-vitro* (DIV). The mouse monoclonal antibody against microtubule-associated protein-2 (MAP-2, CHEMICON International, Inc., Temecula, CA) and the rabbit monoclonal antibody against glial fibrillary acidic protein (GFAP, Santa Cruz Biotechnology, Inc., Santa Cruz, CA) were applied at 1:200 each to stain the neurons and glial cells, respectively. The Texas Red-conjugated goat anti-mouse IgG secondary antibodies (Molecular Probes, Eugene, OR) and fluorescein-conjugated goat anti-rabbit IgG were applied at 10 μg/ml each to respectively recognize the MAP-2 and GFAP primary antibodies.

The sex of the rat E-18 embryos used for primary cortical cultures could be male or female and was not further determined prior to dissection of brain cortices. Similarly, the body weight information of the embryos was unknown. Primary neuronal culture was conducted on a weekly basis, sacrificing approximately 40 pregnant SD rats, each producing approximately 8-16 embryos. The total numbers of rat embryos used for the present study were estimated to be around 8×40=320 to 16×40=640.

Determination of the Molecular Weights and Monosaccharide Compositions of PPV-6

To determine the molecular weights, the PPV-6 samples were first dissolved to 1 mg/mL in 0.1 M $NaNO_3$ (Sinopharm, Beijing, China) with 0.02% NaN_3 prior to filtration through a filter of 0.45-μm. The molecular weights of various fractions were determined by size-exclusion chromatography (SEC) with multi-angle laser light scattering (MALLS)/refractive index (RI), or SEC-MALLS/RI. The weight-average molecular weight (Mw), the number-average molecular weight (Mn), and the polydispersity index (Mw/Mn) were measured on a DAWN HELEOS-II laser photometer (Wyatt Technology Co., Santa Barbara, CA, USA) equipped with two tandem columns (300 × 8 mm, Shodex OHpak SB-805 and 803; Showa Denko K.K., Tokyo, Japan); the temperature was maintained at 45°C by a model column heater from Sanshu Biotech. Co., LTD (Shanghai, China), with the flow rate at 0.6 mL/min. A differential refractive index detector (Optilab T-rEX, Wyatt Technology Co.) was connected simultaneously to give the fraction concentrations and the dn/dc value; the latter was determined to be 0.141 mL/g. Data acquisition and processing were conducted by using ASTRA6.1

(Wyatt Technology Co.).
To determine the monosaccharide compositions, methanol (Cat. No. 67-56-1) and trifluoroacetic acid (Cat. No. 76-05-1) were purchased from ANPEL Laboratory Technologies Inc. (Shanghai, China). Sodium hydroxide (NaOH; Cat. No. 1310-73-2) and sodium acetate trihydrate (NaAc; Cat. No. 127-09-3) were from Sigma (St. Louis, MO, USA). All the standards for monosaccharides were also from Sigma. Five-mg sample was hydrolyzed with trifluoroacetic acid (2 M) at 121°C for 2 h in a sealed tube before blow-drying with nitrogen. Subsequently, the samples were subjected to three washes with methanol before blow-drying. The dried residue was then re-dissolved in deionized water and filtered through a 0.22-µm filter before analysis by high-performance anion-exchange chromatography (HPAEC) on a CarboPac PA-20 anion-exchange column (3 by 150 mm; Dionex) using a pulsed amperometric detector (PAD; Dionex ICS 5000+ system). The parameters were as follows: flow rate, 0.5 mL/min; injection volume, 5 µL; solvent system A: ddH_2O, solvent system B: 0.1 M NaOH, solvent system C: 0.1M NaOH, 0.2M NaAc; gradient program, volume ratio of solution A, B, C was 95:5:0 at 0 min, 85:5:10 at 26 min, 85:5:10 at 42 min, 60:0:40 at 42.1 min, 60:40:0 at 52 min, 95:5:0 at 52.1 min, and 95:5:0 at 60 min. Data were acquired on the ICS5000+ (Thermo Scientific, Waltham, MA, *USA*) and processed using Chromeleon 7.2 CDS (Thermo Scientific).

Experimental designs

Depending on the types of experiments, primary cortical neurons were seeded in 96-well culture plates (MTT reduction assay), coverslips in 24-well culture plates (Hoechst staining and immunocytochemistry), or 6-well culture plates (Western blotting). For co-treatment paradigm, cortical neurons were treated with Aβ (10 µM Aβ25-35 or 5 µM Aβ1-42), PPV-6 at indicated concentrations (1-500 µg/ml), or both for 8-48 h, as denoted in the corresponding figure legends. Control cultures were not additionally treated with equal volumes of vehicles because both Aβs and PPV-6 were dissolved in autoclaved ddH_2O. For post-treatment paradigm (Figure 6), cortical neurons were treated with 10 µM Aβ25-35 for 2 h to trigger neuronal CCR and apoptosis followed by exposure to 250 µg/ml PPV-6, without Aβ25-35, for additional 22 h before subsequent experiments. For SHH-N experiments, cortical neurons were treated with 300 ng/ml SHH-N, 250 µg/ml PPV-6, or both for 24 h before subsequent experiments.

Cell survival assays: MTT reduction assay

The MTT was dissolved in dimethyl sulfoxide (DMSO, Sigma-Aldrich) at 50 mg/ml as a 100-fold stock solution. At the end of drug treatment, C6 glial cells were incubated in culture medium with 0.5 mg/ml MTT at 37°C for 4 h. At the end of 4-h incubation, equal volume of lysis buffer (10% SDS with 0.01 N HCl) was added into the wells and the samples were incubated at 37°C overnight to completely dissolve the formazan precipitates. The absorbance at wavelength 570 nm was determined by the plate reader (SpectraMax® iD3 multi-mode microplate reader, Molecular Device, Sunnyvale, CA, USA).

Cell survival assays: Hoechst staining

For Hoechst staining, cells grown on coverslips were washed with 1× PBS and then fixed with 4% formaldehyde in PBS for 20 min. After several washes, 2.5 µg/ml of Hoechst 33258 was applied to each coverslip. The cells were protected from light and incubated under room temperature for 10 min. After one wash in PBS, the coverslips were mounted on the microscopic slides with mounting medium Vectashield (Vector,

Burlingame, CA, USA) and then stored at 4°C after sealed with nail polish to prevent from dryness. Samples were analyzed under Olympus BX61 fluorescent microscope. The images were acquired with the UV-2A filter. For quantification of cell survival, four visual fields were randomly selected in each experimental condition for counting the numbers of surviving cells with normal nuclear morphology.

Mitochondrial bioenergetics

To determine the bioenergetics of mitochondria, the cellular oxygen consumption rate (OCR) was measured by a XF24 Extracellular Flux Analyzer (Seahorse Bioscience, Billerica, MA) [1-4]. Primary cortical cultures were seeded at a density of 100,000 cells per well on Seahorse XF-24 plates coated with poly-D-lysine and cultured as described above. Subsequent to experimental manipulations, the culture medium was replaced with pre-warmed DMEM medium (Cat. No. 12800, Life technology; DMEM base medium supplemented with 25 mM glucose, 1 mM sodium pyruvate, 110 mM NaCl, 4 mM L-glutamine, pH 7.4) and incubated at 37°C in a non-CO_2 incubator for 1 h before the extracellular flux analysis [1]. Mitochondrial function was quantitatively assessed with sequential injections of oligomycin (the ATP synthase inhibitor), FCCP (the proton ionophore), and antimycin A (the mitochondrial complex III inhibitor), each was diluted in pre-warmed DMEM medium with the final concentration at 1 μM. Four baseline measurements of OCR were determined before injection of mitochondrial inhibitors, and three OCR readings were recorded after addition of each of the above inhibitors. The parameters as indices of mitochondrial function were automatically recorded and calculated by the Seahorse XF-24 software.

Statistical analysis

Results are expressed as Mean $\pm$ S.E.M. from the sample numbers (*N*). For counting of Hoechst-stained surviving cells, the *N* represents data collected from one independent experiment using one cortical culture; each independent experiment contained 3 coverslips for every experimental condition. On each coverslip, 3-4 vision fields were randomly selected for counting to obtain the average numbers of surviving cells in each vision field. The mean numbers of surviving cells for each independent experiment were therefore derived from a total of 9-12 vision fields out of 3 coverslips for the same experimental condition. For Western blotting showing quantitative results, each *N* represents cellular proteins collected from one culture in one experiment; combined data from at least 3 independent experiments using 3 different cultures are shown. For the double immunofluorescence staining, representative images from 3 independent experiments using 3 different cultures are shown. For quantitative determination of OCR to assess mitochondrial functions, combined results derived from 4-5 measurements out of 4-5 different cultures are shown. Multiple groups were analyzed by one-way analysis of variance (ANOVA) followed by a *post-hoc* Tukey test; two groups were analyzed by Student's t-test. P-values of less than 0.05 were considered significant.

References

1. Li, L.; Nadanaciva, S.; Berger, Z.; Shen, W.; Paumier, K.; Schwartz, J.; Mou, K.; Loos, P. et al Human A53T alpha-synuclein causes reversible deficits in mitochondrial function and dynamics in primary mouse cortical neurons. *PLoS One* **2013**, 8, e85815.

2. Giordano, S.; Dodson, M.; Ravi, S.; Redmann, M.; Ouyang, X.; Darley Usmar, V.M.; Zhang, J. Bioenergetic adaptation in response to autophagy regulators during rotenone exposure. *J. Neurochem.* **2014**, 131, 625-633.
3. Im, A.R.; Chae, S.W.; Zhang, G.J.; Lee, M.Y. Neuroprotective effects of Psoralea corylifolia Linn seed extracts on mitochondrial dysfunction induced by 3-nitropropionic acid. *BMC Complement. Altern. Med.* **2014**, 14, 370.
4. Dranka, B.P.; Benavides, G.A.; Diers, A.R.; Giordano, S.; Zelickson, B.R.; Reily, C.; Zou, L.; Chatham, J.C. et al Assessing bioenergetic function in response to oxidative stress by metabolic profiling. *Free Radic. Biol. Med.* **2011**, 51, 1621-1635.
